# Surgical pericardial drainage procedures have a limited diagnostic sensitivity

**DOI:** 10.1111/jocs.14337

**Published:** 2019-11-12

**Authors:** Lindsay Volk, Leonard Y. Lee, Anthony Lemaire

**Affiliations:** ^1^ Division of Cardiothoracic Surgery, Department of Surgery Rutgers Robert Wood Johnson Medical School New Brunswick New Jersey

**Keywords:** drainage, pericardial effusion, tamponade

## Abstract

**Purpose:**

Cardiothoracic surgeons are frequently called upon to perform surgical pericardial drainage procedures (pericardial window) for pericardial effusions. These procedures have therapeutic value, but the diagnostic value of such procedures is debated. We set out to determine the sensitivity of pericardial drainage to detect the disease when cytology, microbiology, and pathology are evaluated.

**Methods:**

A retrospective chart review of patients who underwent pericardial windows from 1 July 2011 to 1 January 2018 at a single academic institution was conducted. All patients who had undergone a recent trauma or cardiac procedure were excluded. Cytology, microbiology, and pathology were examined. The charts were then carefully reviewed to determine if a clinical diagnosis was reached. Sensitivity was then calculated for all diseases and for those that should have been able to be detected.

**Results:**

One hundred sixty‐two patients who had undergone a pericardial drainage procedure were identified; 49 patients were excluded for recent cardiac procedure or trauma. Of the 113 patients who met our inclusion criteria, 56 patients (49.6%) were female with a mean age of 59.7 ± 15.1 years. A diagnosis based on the pathology, microbiology, or cytology was obtained for 27 patients. The most common pathologies detected were adenocarcinoma (11), bacteremia (9), and small cell lung cancer (3); 56 patients had underlying pathologies that would have been possible to detect with either pathology, microbiology, or cytology. The most common detectable diagnoses were adenocarcinoma (20), bacteremia (12), and lymphoma (7). The most common undetectable diagnoses were idiopathic (17), cardiorenal fluid overload (17), and viral (11). The sensitivity of a pericardial drainage procedure for detecting disease was 0.24 for all cases, and 0.48 when restricted to cases where a detectable disease was present.

**Conclusion:**

Cytology, microbiology, and pathology for pericardial drainage procedures were unable to detect a diagnosis for 76% of all cases and greater than 50% of cases with the theoretically detectable disease. Pericardial drainage procedures have a clear therapeutic value, but they have limited diagnostic utility.

## 1 BACKGROUND

The clinical spectrum of patients with pericardial effusions (PEs) due to systemic disease ranges from mild asymptomatic effusions to cardiac tamponade.^1^, ^2^ The treatment options for these patients vary from conservative management with monitoring of the effusions to emergency surgical drainage procedures (pericardial window). There is infrequent data from the literature to guide surgeons in the management of pericardial diseases.^2^ Currently, management is guided by the hemodynamic impact, size, presence of inflammation (ie, pericarditis), and the etiology. Drainage of a PE is required for cardiac tamponade, symptomatic moderate to large PEs, and when a bacterial or neoplastic etiology is suspected.^1^ Both pericardial windows and percutaneous catheter drainage procedures (pericardiocentesis) are options for removing the PE. The consensus from the literature supports pericardiocentesis when there is hemodynamic instability because of how quickly it can be done and it does not require going to the operating room.^3^ Although a pericardiocentesis has the advantage of being less invasive and more efficient, surgical drainage procedures have several advantages. First, surgical drainage procedures have decreased rates of recurrence reported compared with a pericardiocentesis.^4^ Second, pericardial tissue can be sampled as well allowing for more diagnostic options for the PE. The potential etiology of PEs are varied and include idiopathic, neoplastic, infectious, related to connective tissue diseases, metabolic, and iatrogenic.^5^ As a result, surgical drainage can be both therapeutic and diagnostic.^6^, ^7^, ^8^ The diagnostic yield of the samples from a pericardial drainage procedure is controversial.^5^ The value of the pathology findings from this tissue is not well established,^4^, ^9^ although pericardial biopsy may be indicated in patients with persistent worsening illness without a definite diagnosis.^5^ Previous studies have shown that the diagnostic success of pericardial windows is limited, but the sensitivity of this procedure has not yet been assessed outside of cohorts of only patients with malignancy.^7^ The objective of the study is to evaluate the sensitivity of pericardial windows to make a clinical diagnosis.

## 2 METHODS

A retrospective chart review of patients who underwent surgical drainage procedures of PEs from 1 July 2011 to 1 January 2018 at a single academic institution was conducted. Patients undergoing a surgical pericardial drainage procedure were selected. Demographic information was collected for each patient. Preoperative, intraoperative, and postoperative findings were identified through a thorough review of the electronic medical record. Postoperative complications, morbidity, and survival information were also reviewed. In each operative case included in this study, at least 50 cc of pericardial fluid was sent to the microbiology and cytology laboratories for analysis. A large sample of the pericardium was also sent to the pathology laboratory for testing. Patients who had recently undergone a prior cardiac procedure or experienced trauma were excluded. This distinction was made by reviewing admission and prior procedural documentation. Hospital records and laboratory findings were reviewed to determine a final diagnosis when available. This study was approved by the institutional review board at Rutgers Robert Wood Johnson Medical School.

## 3 OPERATIVE TECHNIQUES

The technique for surgical drainage of PEs is primarily through a subxiphoid or thoracotomy approach. When the PE is drained through the subxiphoid approach a 3 cm incision is made at the level of the xiphoid. Electrocautery is then used to divide the subcutaneous tissue down to the xiphoid process. Next, a combination of sharp and blunt dissection is used to isolate and resect the xiphoid. The remaining sternum is elevated with a retractor and the pericardium is identified. The pericardial space is entered, and the effusion is drained. The opening of the pericardium is enlarged to ensure patency and drainage. At the completion of the procedure, a drain is placed within the pericardium and secured. Similarly, the pericardium can be approached by going through either the left or right pleural space. Once the pericardium is identified it is entered and the effusion drained. The method of entering the pleural space can be through an open thoracotomy or with a video‐assisted thoracoscopic approach.

## 4 RESULTS

**Figure 1:**
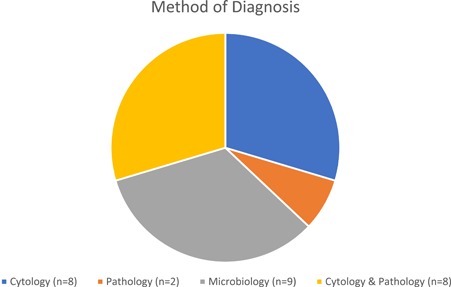
Number of pericardial drainage procedure based diagnosis by the method

There were 162 patients who had undergone a surgical pericardial drainage procedure identified, 49 patients were excluded owing to recent cardiac procedure or trauma. Of the 113 patients who met our inclusion criteria, 56 patients (49.6%) were female and the average age was 59.7 ± 15.1 years. A diagnosis based on the pathology, microbiology, or cytology was obtained for 27 patients (23.9%). The most commonly returned diagnosis was adenocarcinoma which was found in 11 patients (9.7%), followed by bacteremia in 9 patients (8.0%), and small cell cancer in 3 (2.7%). Melanoma, mesothelioma, and other malignancies made up the remainder. These are detailed in Table 1. The diagnosis was achieved by cytology alone in 8 patients (29.6%), by microbiology in 9 patients (33.3%), pathology alone in 2 patients (7.4%), and by both cytology and pathology in 8 patients (29.6%) (Figure 1).

**Table 1:** Diagnoses detected by pericardial drainage procedure

Diagnosis	Number (% of total patients)
Adenocarcinoma	11 (9.7)
Bacteremia	9 (8.0)
Small Cell	3 (2.7)
Melanoma	1 (0.9)
Mesothelioma	1 (0.9)
Other Malignancies	2 (1.8)
Total	27 (23.9)

On the basis of the chart review, 56 patients had underlying pathologies identified that would have been possible to detect with either pathology, microbiology, or cytology (Table 2). The most common detectable diagnoses were adenocarcinoma in 20 patients (17.7%), bacteremia in 12 patients (10.6%), and lymphoma in 7 patients (6.2%). Other diagnoses included leukemia, small cell, melanoma, and mesothelioma. Fifty‐five percent of the adenocarcinoma and 75% of the bacteremia cases were able to be detected. Similarly, 57 patients had diagnoses that were unlikely to be detected using cytology, pathology, or microbiology. The most common undetectable diagnoses were idiopathic in 17 patients (15.0%), cardiorenal fluid overload in 17 patients (15.0%), and viral in 11 patients (9.7%) (Table 3). The sensitivity of a pericardial drainage procedure for detecting disease was 0.24 for all cases included in this series, and 0.48 when restricted to cases where a detectable disease was present.

**Table 2:** Incidence of diseases that may have potentially been detected on pericardial drainage procedure

Diagnosis	Number (% of total patients)
Adenocarcinoma	20 (17.7)
Bacteremia	12 (10.6)
Lymphoma	7 (6.2)
Leukemia	6 (5.3)
Small cell	4 (3.5)
Melanoma	2 (1.8)
Mesothelioma	1 (0.9)
Other malignancies	4 (3.5)
Total	56 (49.6)

**Table 3:** Incidence of diseases that were unlikely to be detected on pericardial drainage procedure

Diagnosis	Number (% of total patients)
Unknown	17 (15.0)
Fluid overload (ESRD, CHF, cardiorenal syndrome)	17 (15.0)
Viral	11 (9.7)
Autoimmune	7 (6.2)
Chronic pericarditis	2 (1.8)
Benign asbestos	1 (0.9)
Amyloidosis	1 (0.9)
Uremia	1 (0.9)
Total	57 (50.4)

## 5 DISCUSSION

Surgical drainage of PEs allows for the removal of fluid which may lead to tamponade physiology and potential mortality. The therapeutic valve of pericardial drainage procedures has been well demonstrated both in the literature and in daily clinical practice. The diagnostic value of such procedures is still very much debated.^5^, ^6^, ^7^, ^8^ These findings show that while pericardial windows could provide additional information when cytology, microbiology, and pathology are employed, the sensitivity of this information is relatively low. The sensitivity of a pericardial drainage procedure for detecting disease was 0.2 for all cases included in this series, which correlates to a diagnosis rate of 1 in 5. This is slightly improved to a sensitivity of 0.48 when considering only diseases that should have been captured on cytology, microbiology, and pathology. Acceptable thresholds for the sensitivity of this procedure need to be set by individual clinicians, based on individual patients, but these findings should help guide that decision. It is reasonable to conclude that if a pericardial window is required for its therapeutic value then the diagnostic value is an added benefit, but with a sensitivity of only 0.2, it is difficult to recommend a pericardial window solely as a diagnostic procedure.

This is one of the few studies to look at a large population of patients who underwent surgical pericardial drainage procedures to assess the diagnostic value of the procedure. Moreover, our analysis also is unique in that we assessed the sensitivity in the subset of patients that actually had a diagnosis such as malignancy. In a study looking at patients with a known malignancy that had undergone a pericardial drainage procedure there were positive cytology findings in 44% and positive pathology findings in 24%.^7^ This study was able to diagnosis adenocarcinoma in 55% of the cases. It is reassuring that our study findings agree with the existing literature. Analyzing subsets of patients has its value, but in cases where the underlying etiology is unknown, it is important to consider the sensitivity for all patients.

Furthermore, it is not surprising that pathology alone found the diagnosis in only two patients. Prior studies have found that the addition of pericardial tissue examination added little to the cytologic evaluation of the pericardial fluid.^4^, ^9^ Pericardial metastases are a rare finding but occur most commonly with lung cancer, breast cancer, and lymphoma and leukemia.^10^ Despite the low yield of finding cancer within the pericardial sample sent, the removal of tissue for pericardial windows is inherent to the procedure. As a result, sending the pericardial tissue for analysis does not add to the risk of the procedure and should be continued.

It is important to address the limitations of this study. It is a retrospective chart review from a single institution and is subject to the biases and issues of generalizability that are inherent to the study design. It is also possible that the distribution of disease in the study population impacted the findings, although the distribution is in agreement with existing studies. In other published studies, a neoplastic origin of PE ranged from 15% to 23%, infectious from 2% to 27%, and idiopathic in 7% to 48%.^11^, ^12^, ^13^ Additional studies in other populations will be required to further validate these findings. These findings are also subject to the limitations inherent in the cytology, microbiology, and pathology process. It is likely that as the sensitivity of these methods increases, so will the sensitivity for fluids and tissues collected during pericardial windows.

Finally, pericardial drainage procedures, when cytology, microbiology, and pathology are employed, are unable to detect a diagnosis for 76% of all cases and greater than 50% of cases with the theoretically detectable disease. Pericardial drainage procedures have a clear therapeutic value, but they should be used with caution for solely diagnostic reasons as the sensitivity is relatively low.
